# Supplementary material for: Electrochemically Reduced Graphene Oxide Covalently Bound Sensor for Paracetamol Voltammetric Determination
Source: Int J Mol Sci. 2025 Apr 30;26(9):4267. doi: 10.3390/ijms26094267 (PMC12072182; doi:10.3390/ijms26094267)
Supplement: Supplementary file 1 [file ijms-26-04267-s001.zip › ijms-3571167-supplementary.pdf]

International Journal of
Molecular Sciences

MDPI

Supplementary information

Electrochemically Reduced Graphene Oxide Covalently Bind Sensor for Paracetamol Voltammetric Determination

Amaya Paz de la vega [1], Fabiana Liendo [1], Bryan Pichún [1,2], Johisner Penagos [1], Rodrigo Segura [1,*] and Maria Jesús Aguirre [1,2,*]

[1] Department of Chemistry of Materials, Faculty of Chemistry and Biology, Universidad de Santiago de Chile (USACH), Santiago, Chile.

[2] Millennium Institute on Green Ammonia as Energy Vector—MIGA (ICN2021_023), Santiago, Chile.

Contents:

1. **Figure S1:** Electroactive surface area.
2. **Figure S2:** Optimization of the electrografting process.
3. **Figure S3:** Optimization of modified GO/NaNO2/HCl agent.
4. **Figure S4:** Optimization of supporting electrolyte.
5. **Figure S5:** Acid-Base equilibrium of PAR in aqueous solution.
6. **Figure S6:** Excipient analysis.

1. Electroactive Surface Area

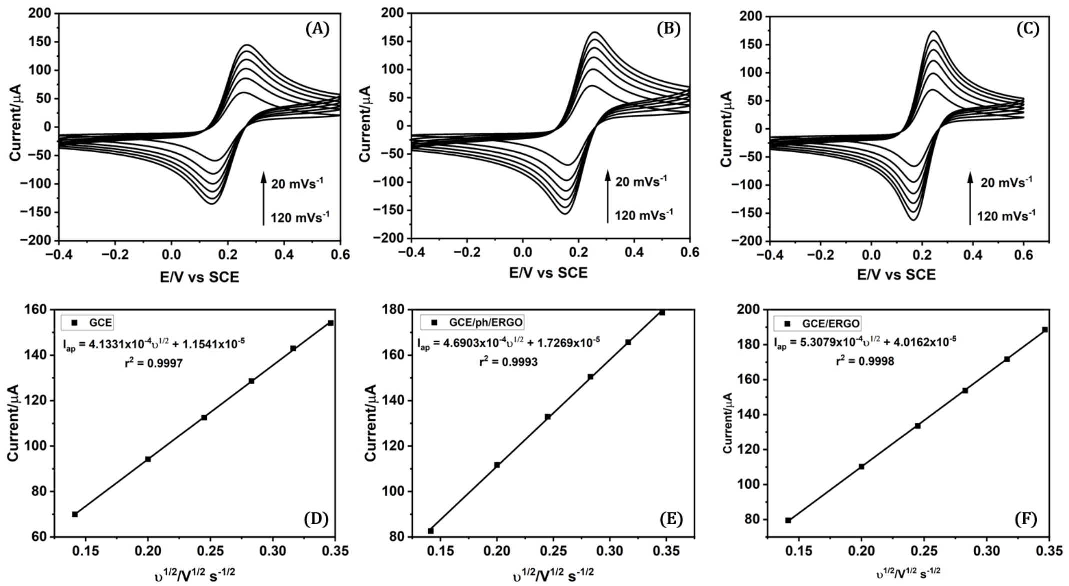

Figure S1. Cyclic voltammograms obtained using different scan rates (20 – 120 mV s^{-1}) for the redox probe and the corresponding representation of the current versus square root of the scan rate, with the respective equations and Pearson correlation coefficient using (A - D) GCE, (B - E) GCE/ph/ERGO, and (C - F) GCE/ERGO in 5 mM $Fe(CN)_6^{3-/4-}$ 0.10 M KCl and 0.10 M PBS pH 7, range -0.40 to 0.60 V.

2. Optimization of the Electrografting Process

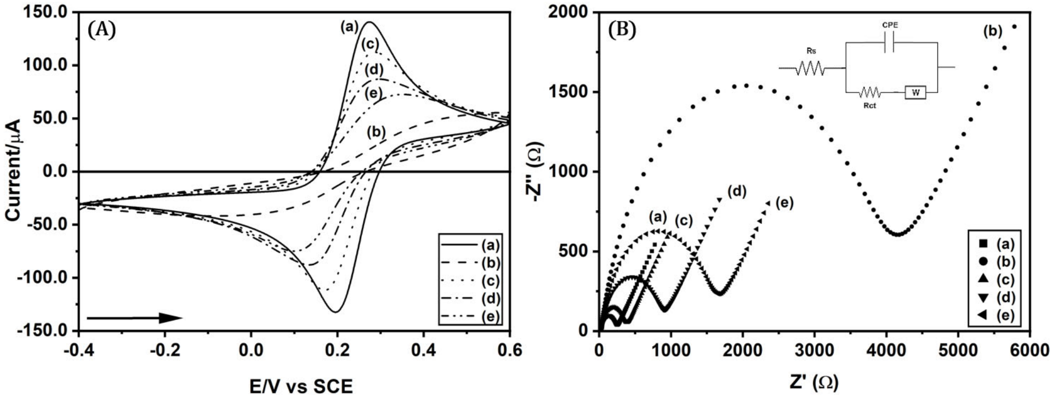

Figure S2. A. Cyclic voltammograms and B. Nyquist plots for (a) GCE, (b) GCE/pNO$_2$, (c) GCE/pNH$_2$ (1 mM pNA: 1 mM NaNO$_2$), (d) GCE/pNH$_2$ (1 mM pNA: 2 mM NaNO$_2$), and (e) GCE/pNH$_2$ (1 mM pNA : 3 mM NaNO$_2$) in 5 mM Fe(CN)$_6^{3-/4-}$ 0.10 M KCl and 0.10 M PBS pH 7, range -0.40 to 0.60 V, 100Mv s^{-1}, scanning in a frequency range from 0.10 to 100,000 Hz. Inset: Electrical circuit used to process EIS spectra.

3. Optimization of Modified GO/NaNO2/HCl Agent

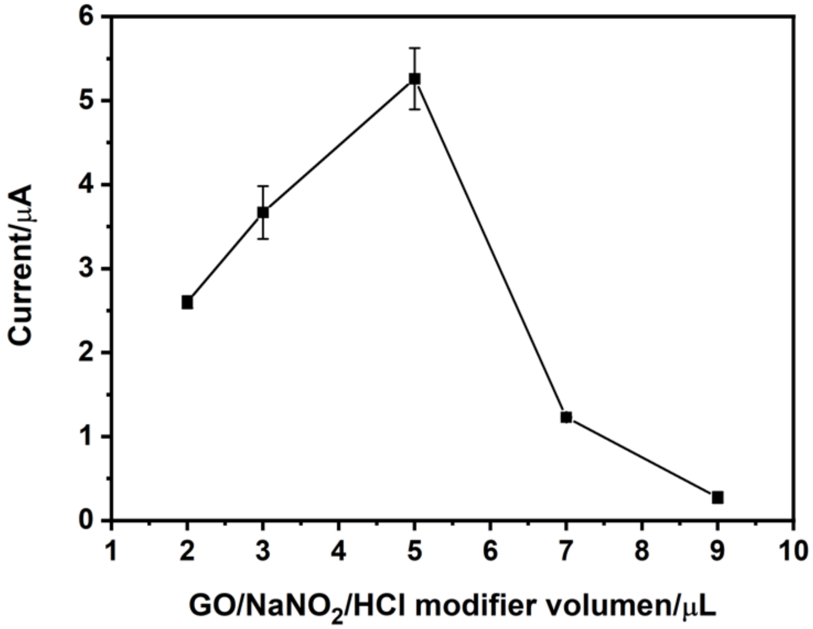

Figure S3. Current dependence of PAR 2 μM on the modifier GO/NaNO$_2$/HCl volume. Conditions: n = 3, scanning range 0.3 – 0.7 V, 0.1 mol L^{-1} acetate buffer (pH 5).

4. Optimization of SUPPORTING electrolyte

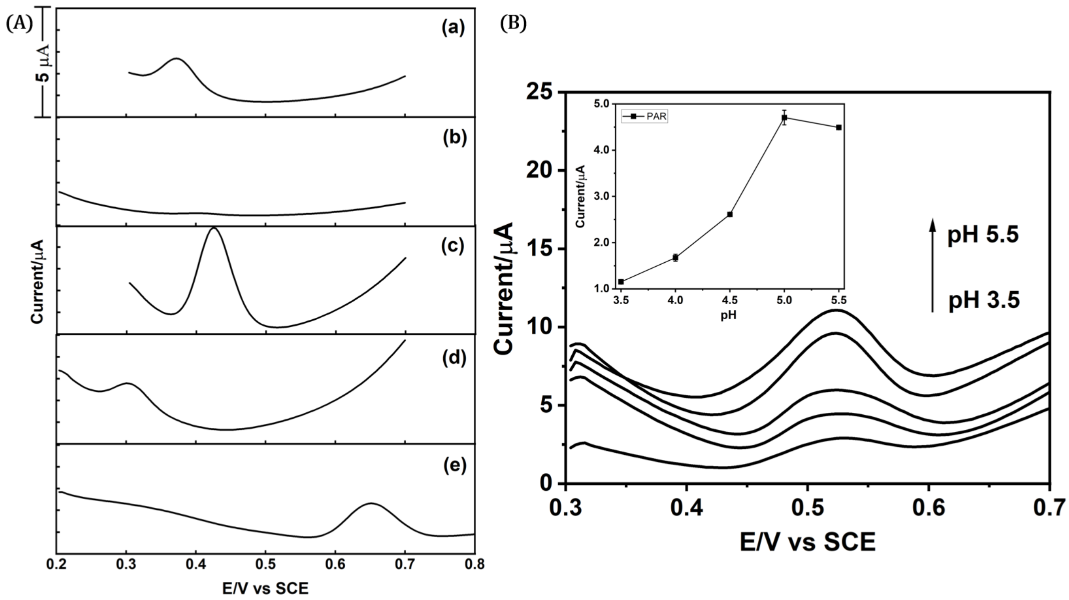

Figure S4. SWV measurements of PAR 2 μM **A.** performed in the presence of different buffers: (a) 0.1 mol L^{-1} phosphate buffer (pH 7), (b) 0.1 mol L^{-1} Britton Robinson buffer (pH 6.3), (c) 0.1 mol L^{-1} acetate buffer (pH 5), (d) 0.1 mol L^{-1} ammonia buffer (pH 8.5), and (e) 0.1 mol L^{-1} sulfuric acid, and **B.** performed in different pH's of acetate buffer (0.1 mol L^{-1}). Inset: Effect of acetate buffer pH's on 2 μM PAR current.

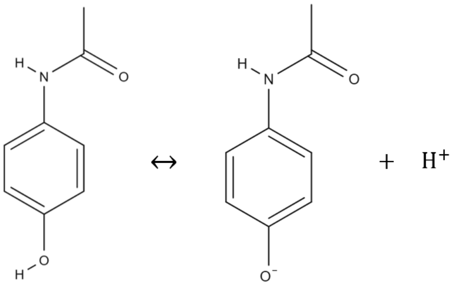

PAR
pK_a 9.46

Figure S5. Acid-Base equilibrium reaction of PAR in aqueous solution.

5. Excipient Analysis

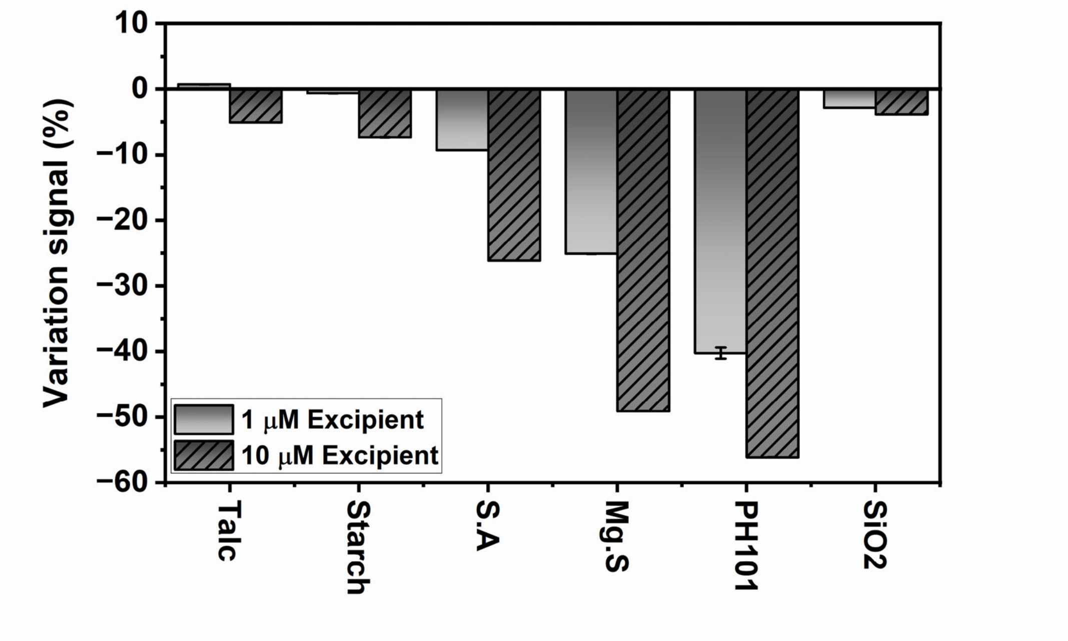

Figure S6. Interference of excipients on the peak current of 0.1 µM PAR under optimized conditions. **S.A:** stearic acid and **Mg.S**: magnesium stearate.
